# Case Report: Durable response to immuno-chemotherapy in a case of ROS1 fusion-positive advanced lung adenocarcinoma: A case report

**DOI:** 10.3389/fphar.2022.898623

**Published:** 2022-09-09

**Authors:** Ningning Yan, Siyuan Huang, Linlin Li, Qianqian Guo, Di Geng, Huixian Zhang, Sanxing Guo, Xingya Li

**Affiliations:** Department of Medical Oncology, The First Affiliated Hospital of Zhengzhou University, Zhengzhou, China

**Keywords:** immune checkpoint inhibitor, NSCLC, ROS1, chemo-immunotherapy, lung adenocarcinoma

## Abstract

Immune checkpoint inhibitors (ICIs) have greatly transformed the treatment and improved the prognosis for patients with non-small cell lung cancer (NSCLC) without driver gene alterations. However, the effects of ICI combination therapy in ROS1 fusion-positive NSCLC remains unclear. Herein, we present a case with ROS1 fusion-positive NSCLC treated with ICI plus chemotherapy. The patient achieved a continuous partial response (PR) to ICI plus chemotherapy and a more than 35 months progression free survival. This case demonstrates that ICI plus chemotherapy is a promising option for patients with ROS1 fusion-positive NSCLC.

## Introduction

Current standard treatment recommendations in USA and Europe for ROS1 fusion-positive non-small cell lung cancer (NSCLC) are ROS1-tyrosine kinase inhibitors (TKIs), including crizotinib or entrectinib, which result in an overall response rate of 65–77% and a progression-free survival (PFS) of approximately 16–19 months (Wu et al., 2018; Moro-Sibilot et al., 2019; Shaw et al., 2019; Drilon et al., 2020). However, the only ROS1-TKI available in China was crizotinib because of the unavailability of entrectinib.

Recently, immune checkpoint inhibitors (ICIs) have greatly changed the treatment options for driver mutation-negative advanced NSCLC. However, whether immunotherapy is effective for treating ROS1 fusion-positive advanced-stage NSCLC has not been widely examined. An IMMUNOTARGET registry study that included 24 centers in 10 countries was conducted to investigate the efficacy of ICI in NSCLC with driver gene alterations; the results showed that patients treated with ICI monotherapy achieved an overall response rate of 16.7% (Mazieres et al., 2019). However, the effectiveness of ICI combined with chemotherapy in patients with ROS1 alterations in advanced NSCLC warrants further investigation. Herein, we report a case of a TKI-naïve, non-smoking female diagnosed with stage IV lung adenocarcinoma with CD74-ROS1 fusion concomitant with negative PD-L1 expression. The patient was treated with pembrolizumab in combination with chemotherapy, providing a new perspective for the treatment of ROS1-altered lung cancer.

## Case description

**FIGURE 1:**
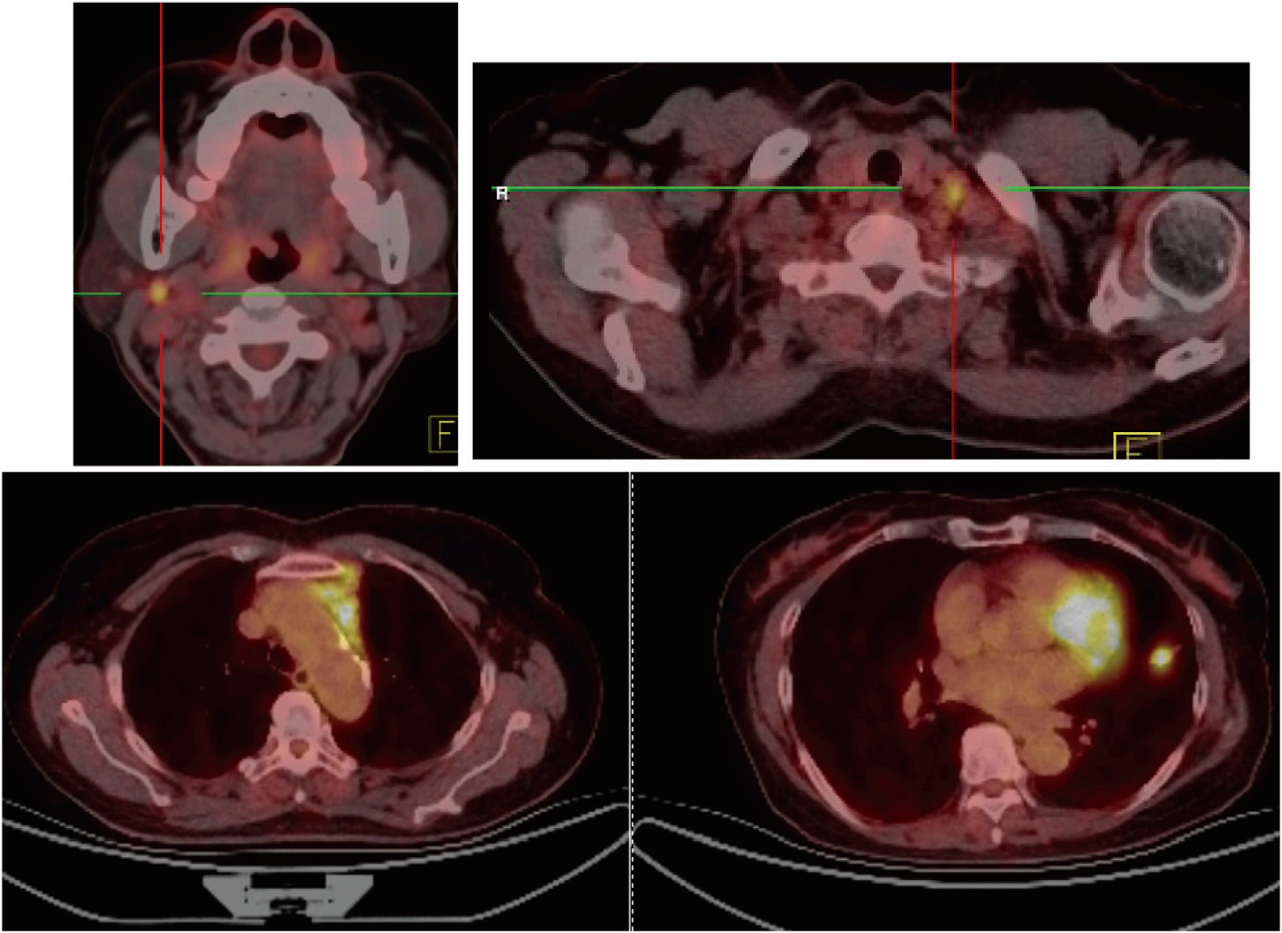
Positron emission tomography/computed tomography imaging. Positron emission tomography/computed tomography imaging on 19 February 2019 revealed an enlarged nodule measuring 1.4 cm × 1.5 cm on let upper lobe (LLL) with a maximum standardized uptake value (SUVmax) of 8.6, right cervical lymph nodes at 0.9 cm × 1.0 cm with SUVmax 8.8.

A 64-year-old female non-smoker with progressive pain in both legs for 3 months was admitted to our hospital on 10 February 2019. Positron emission tomography/computed tomography scans showed a lung nodule of 14 mm × 15 mm in the left upper lobe with right cervical, left supraclavicular, left mediastinal, and left internal mammary lymph node metastases. The patient was clinically diagnosed as stage IV-T1bN3M1 with an Eastern Cooperative Oncology Group performance score of 1 at initial diagnosis (Figure 1). Fine-needle aspiration biopsy on the supraclavicular lymph node revealed metastatic adenocarcinoma, and lung nodule biopsy confirmed PD-L1-negative lung adenocarcinoma. Reverse transcription polymerase chain reaction analysis of the biopsy tumor sample showed that the tumor was EGFR-negative, and the immunohistochemistry results revealed a negative expression of anaplastic lymphoma kinase (ALK). Subsequently, DNA based next-generation sequencing of 425 cancer-related genes using Illumina MiSeq (San Diego, CA, Unied States) on the biopsy tissue samples revealed a CD74-ROS1 gene fusion and tumor mutation burden (TMB) of 12.6 mutations/megabase after one cycle of treatment with pembrolizumab (200 mg day 1) plus carboplatin (600 mg with AUC 4 at day 1) and pemetrexed (800 mg with 500 mg/m^2^ at day 1) (Table 1). This patient was treated with 6 cycles of pembrolizumab plus carboplatin and pemetrexed, followed by 21 cycles of maintenance therapy with pemetrexed and pembrolizumab. Finally, 8 cycles of pembrolizumab monotherapy was administered after 2 years of treatment.

**TABLE 1:** Summary of genomic alterations tested via next-generation sequencing.

Gene	Transcript code	Exon	Result	Abundance (%)	Alteration type
*ROS1*	NM_002944.2	6:34	CD74-ROS1 fusion	10.7	Gene fusion
*CTNNB1*	NM_001904.3	3	p. S37F	19.4	Missense mutation
*KMT2B*	NM_014727.1	35	p. E2575K	4.8	Missense mutation
*MRE11A*	NM_005590.3	4	p. E64K	17.9	Missense mutation
*NF1*	NM001042492	24	p. D1058N	4.2	Missense mutation
*QKI*	NM_006775.2	1	p. L18V	25.5	Missense mutation
*RAD54L*	NM_003579.3	12	p. Q420*	1.3	Nonsense mutation
*SPOP*	NM_003563.3	11	p. S355F	18.1	Missense mutation
*TP53*	NM001126112	5	p. Q144*	22.3	Nonsense mutation
*CD74*	NM_004355	6	IGR (GSTA3)-CD74 fusion	3.0	Gene fusion

**FIGURE 2:**
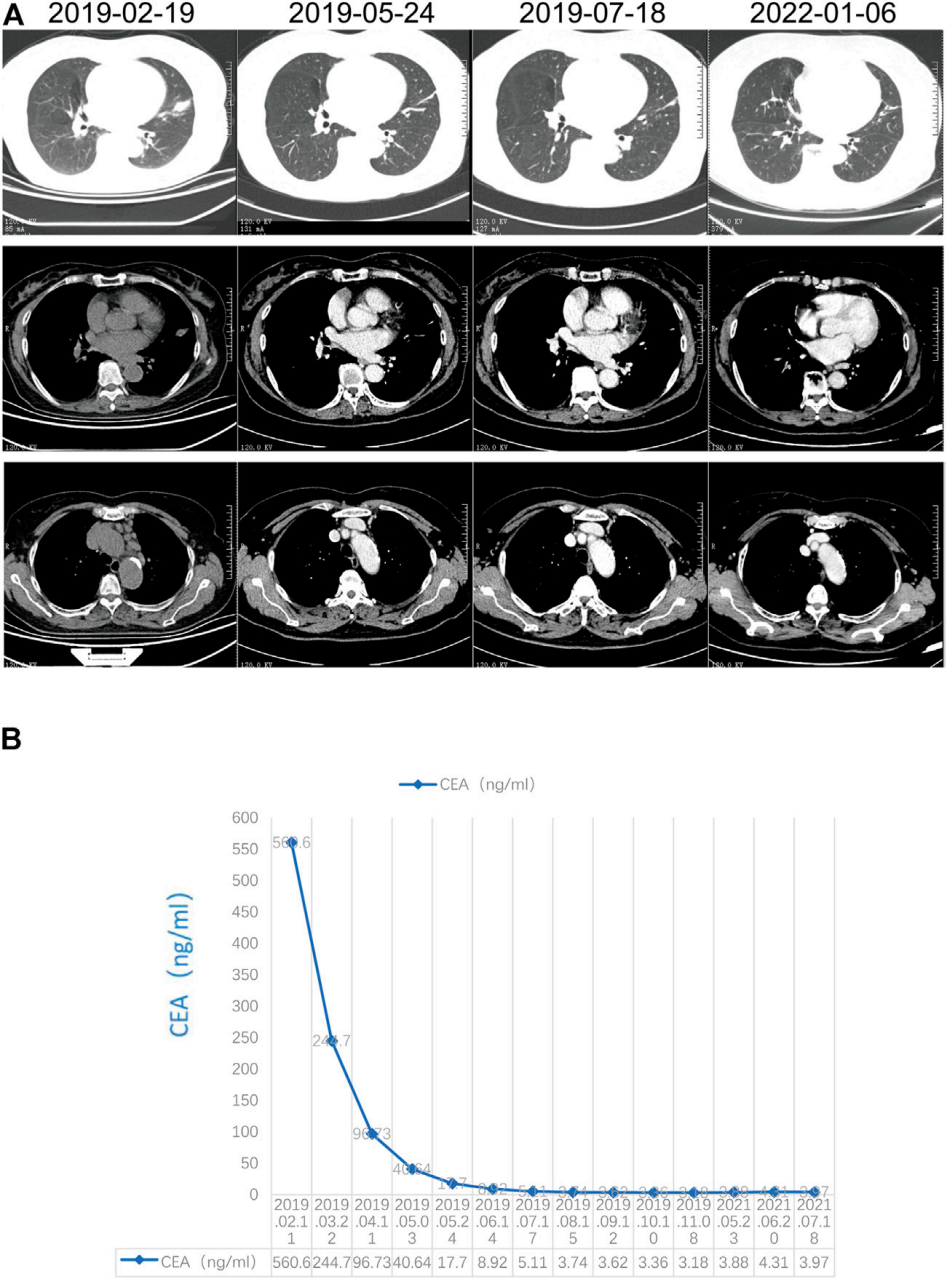
Response evaluation. **(A)** Radiographic response evaluation; **(B)** Change in carcinoembryonic antigen.

Because of the EGFR/ALK negativity and urgency to treat the patient, we selected ICI in combination with chemotherapy based on the recommendations for driver mutation-negative advanced stage NSCLC from the National Comprehensive Cancer Network and Chinese Society of Clinical Oncology guidelines. After the first treatment cycle, the carcinoembryonic antigen levels drastically decreased from 560.60 to 244.70 ng/ml, and the patient’s symptoms were significantly alleviated. Hence, this regimen was continued without switching to ROS1-TKI, although next-generation sequencing revealed a positive CD74-ROS1 fusion alteration. A partial response, including a 60.2% reduction in the primary target lesion and 74.7% reduction in the metastatic lymph nodes, was observed on the first computed tomography scan evaluation after two cycles of treatment with pembrolizumab combined with carboplatin/pemetrexed (Figure 2A). A continued partial response was achieved using pembrolizumab/pemetrexed maintenance, with a PFS of more than 35 months. In addition, carcinoembryonic antigen levels decreased to the normal range (0–5 ng/ml) (Figure 2B). The most common adverse events were hematologic toxicities, including grade 2 leukopenia, grade 1 neutropenia, and grade 1 anemia. In addition, grade 1 liver injury occurred during maintenance therapy with pemetrexed and pembrolizumab.

## Discussion

ROS1 is an oncogene encoding a receptor tyrosine kinase and shows considerable homology with other members of insulin receptor family of receptor tyrosine kinases, particularly ALK (Acquaviva et al., 2009). Hence, ALK inhibitors such as crizotinib exhibit promising anti-tumor activity in ROS1 fusion-positive NSCLC (Wu et al., 2018). In addition, the current standard treatment for ROS1 fusion-positive advanced NSCLC is ROS1-TKIs including crizotinib and entrectinib in USA. In China, because entrectinib is unavailable, the first-line treatment for ROS1 fusion-positive NSCLC is crizotinib. However, the strength of this recommendation is moderate because of the low evidence quality (Wu et al., 2018). In the present study, we report a patient diagnosed with ROS1-altered advanced NSCLC and treated with ICI plus platinum-based chemotherapy; the patient achieved a partial response, and PFS was more than 35 months.

A previous study suggested that higher PD-L1 expression (PD-L1 ≥50%) predicted a good response to ICI monotherapy in driver mutation-negative advanced NSCLC (Reck et al., 2016). However, the latter may benefit from ICI in combination with chemotherapy regardless of PD-L1 expression. In addition, driver gene mutations were considered as negative predictive markers of ICIs. In the IMMUNOTARGET registry study, patients with ROS1 fusion NSCLC displayed an unsatisfactory overall response rate of 16.7% (Mazieres et al., 2019). However, the patients included in this study were treated only with ICI monotherapy. Notably, patients with ROS1 fusion-altered advanced NSCLC showed a high level of PD-L1 expression; in this study, 60% patients presented PD-L1 levels of ≥50% (Mazieres et al., 2019). Recently, another study showed that ROS1 fusion-positive NSCLC harbored higher PD-L1 expression. In addition, this report indicated that ICI monotherapy performed much poorer than ICI combined with chemotherapy, with 2.1 months of time to treatment discontinuation in ICI monotherapy and 10.0 months of time to treatment discontinuation (Choudhury et al., 2021). Hence, according to the results of our study and those of previous reports, ICI in combination with chemotherapy shows potential for treating ROS1 fusion-positive NSCLC. However, in our case, PD-L1 expression was negative, and the TMB level was 12.6 mutations/megabases. A previous study showed that patients with a high tissue TMB (TMB ≥10 mutations/megabase) would benefit from pembrolizumab (Marabelle et al., 2020). This may partially explain why our patient showed a robust response to ICIs. In addition, the next-generation sequencing results revealed other gene mutations, including in *TP53*, which were related to poorer prognosis. Previous studies revealed that pemetrexed-based chemotherapy is more effective for patients with ROS1-altered NSCLC than for those with ROS1 wild-type or oncogenic mutations (such as KRAS mutations) (Drilon et al., 2021). Hence, our data suggest that chemo-immunotherapy can be used in ROS1-altered NSCLC. The selection of a chemo-immunotherapy regimen is useful in cases with negative EGFR/ALK and an unknown fusion status. However, ROS1 TKI therapy remains the preferred treatment option for treatment-naïve patients with ROS1 fusion based on prospective trials showing that ROS1 TKI therapy achieves prolonged overall disease control. Additionally, prospective randomized stage III clinical trials are being conducted to investigate the first line setting of ROS1 fusion-positive NSCLC.

Furthermore, no confirmed evidence supports that ROS1 TKI therapy can be used in post-progression to chemoimmunotherapy. Notably, ICIs sequential with TKI inhibitors may increase the incidence of severe adverse events in EGFR-mutated NSCLC (Schoenfeld et al., 2019). In addition, in a previous study, 453 patients were treated with crizotinib for ALK/ROS1/MET alterations; 5 of 11 (45.5%) patients treated with ICI followed by crizotinib developed grade 3/4 liver enzyme elevation, compared with 8% of those administered crizotinib monotherapy (Calles et al., 2020). Comparatively, TKI followed by ICI appeared to be a safer option.

## Conclusion

We observed a durable response to pembrolizumab in combination with chemotherapy and a prolonged PFS of over 35 months in a treatment-naïve patient with ROS1 fusion-positive and PD-L1-negative lung adenocarcinoma. Thus, chemo-immunotherapy is a promising option for ROS1 fusion-positive NSCLC.

## Data Availability

The datasets presented in this study can be found in online repositories. The names of the repository/repositories and accession number(s) can be found below: https://www.ncbi.nlm.nih.gov; PRJNA836314.
