# Corrigendum: Case Report: Durable response to immuno-chemotherapy in a case of ROS1 fusion-positive advanced lung adenocarcinoma: A case report

**DOI:** 10.3389/fphar.2023.1256801

**Published:** 2023-07-26

**Authors:** Ningning Yan, Siyuan Huang, Linlin Li, Qianqian Guo, Di Geng, Huixian Zhang, Sanxing Guo, Xingya Li

**Affiliations:** Department of Medical Oncology, The First Affiliated Hospital of Zhengzhou University, Zhengzhou, China

**Keywords:** immune checkpoint inhibitor, NSCLC, ROS1, chemo-immunotherapy, lung adenocarcinoma

In the published article, the names in the **Author list** were incorrectly written as “Ning Yan^*†^, Si Huang^†^, Lin Li, Qian Guo, Di Geng, Hui Zhang, San Guo^*^ and Xing Li^*^.” The correct spelling is “Ningning Yan^*†^, Siyuan Huang^†^, Linlin Li, Qianqian Guo, Di Geng, Huixian Zhang, Sanxing Guo^*^ and Xingya Li^*^.” The updated **Author Contributions statement** appears below.

“All authors participated in the diagnosis and treatment process. NY, SH, and XL diagnosed the patient and developed the treatment plan. NY, SH, LL, QG, DG, and HZ participated in the follow-up course. NY, SH, SG, and XL wrote the original manuscript. All authors contributed to the article and approved the submitted version.”

The authors apologize for this error and state that this does not change the scientific conclusions of the article in any way. The original article has been updated.

